# Cognitive frailty in relation to adverse health outcomes independent of multimorbidity in Chinese older adults

**DOI:** 10.1093/geroni/igaa057.3342

**Published:** 2020-12-16

**Authors:** Zuyun Liu, Chen Chen, Juyoung Park, Chenkai Wu, Qian-Li Xue, George Agogo, Ling Han, Zunyou Wu

**Affiliations:** 1 School of Public Health and the Second Affiliated Hospital, Zhejiang University School of Medicine, Hangzhou, China; 2 Chinese Center for Disease Control and Prevention, Beijing, China; 3 Boca Raton, Florida, United States; 4 Duke Kunshan University, Kunshan, Jiangsu, China; 5 Johns Hopkins School of Medicine, Baltimore, Maryland, United States; 6 Centers for Disease Control and Prevention, Nairobi, Kenya; 7 Yale School of Medicine, New Haven, Connecticut, United States

## Abstract

Cognitive frailty was proposed in 2013 by an (I.A.N.A./I.A.G.G.) international consensus group; however, little is known about its status and associations with adverse health outcomes in China. The objectives of this study were to evaluate: 1) the associations of cognitive frailty with various health outcomes including disability, hospitalization, and death; 2) whether the associations differed by multimorbidity in Chinese older adults. We included 5113 Chinese older adults (aged 60+ years) who had baseline (2011 wave) cognition and physical frailty assessments and follow-up for 4 years from the China Health and Retirement Longitudinal Study. We found that about 16.0% had cognitive impairment; 6.7% had physical frailty; and 1.6% met criteria for cognitive frailty (having both cognitive impairment and physical frailty). Both cognitive impairment (odds ratios (ORs) range: 1.41 to 2.11) and physical frailty (ORs range: 1.51 to 2.43) were independently associated with basic activities of daily living (BADL), instrumental ADL (IADL), mobility disability, hospitalization, and death among participants without that corresponding outcome at baseline, even after accounting for covariates. Relative to participants who had normal cognition and were nonfrail, those with cognitive frailty had the highest risk for IADL disability (OR=3.40, 95% CI, 1.23–9.40) and death (OR=3.89, 95% CI, 2.25–6.47). We did not find significant interaction effects between cognitive frailty and multimorbidity (P for interactions>0.05). Overall, cognitive frailty was associated with disability and death, independent of multimorbidity. This highlights the importance of assessing cognitive frailty in the community to promote primary and secondary preventions for healthy aging.

